# Supplementary material for: (−)-Lariciresinol Isolated from the Roots of Isatis indigotica Fortune ex Lindl. Inhibits Hepatitis B Virus by Regulating Viral Transcription
Source: Molecules. 2022 May 18;27(10):3223. doi: 10.3390/molecules27103223 (PMC9143483; doi:10.3390/molecules27103223)
Supplement: Supplementary file 1 [file molecules-27-03223-s001.zip › molecules-1679412-Supplementary.pdf]

Supplementary Material

The structural information and LC-MS analysis of (−)-lariciresinol are as follows:

(−)-lariciresinol was obtained as white amorphous powder. ^{1}H NMR (CD_3OD, 500 MHz) δ 6.85 (d, J = 1.5 Hz, 1H), 6.71 (m, 2H), 4.69 (d, J = 7.0 Hz, 1H), 2.32 (m, 1H), 3.78 (1H), 3.57 (dd, J = 11.0, 3.5 Hz, 1H), 6.74 (d, J = 1.5 Hz, 1H), 6.65 (m, 1H), 6.58 (dd, J = 8.0, 1.5 Hz, 1H), 2.87 (dd, J = 13.5, 4.5 Hz, 1H), 2.43 (dd, J = 11.0, 13.5 Hz, 1H), 2.67 (m, 1H), 3.92 (dd, J = 8.0, 6.0 Hz, 1H), 3.67 (dd, J = 8.0, 4.0 Hz, 1H), 3.78 (3H), 3.77 (3H); ^{13}C NMR (CD_3OD, 125 MHz) δ 137.5, 110.5, 149.0, 147.0, 116.0, 119.8, 84.0, 54.1, 60.4, 133.5, 113.3, 149.0, 145.8, 116.2, 122.2, 33.6, 43.9, 73.5, 56.3; HRMS: calcd for $C_{20}H_{24}O_6Na$ [M + Na]$^+$: 383.1462, found: 383.1465.

Thermo Qexactive Focus Report

compound **MI-54a**
Method : **LCMS(compound)-low**

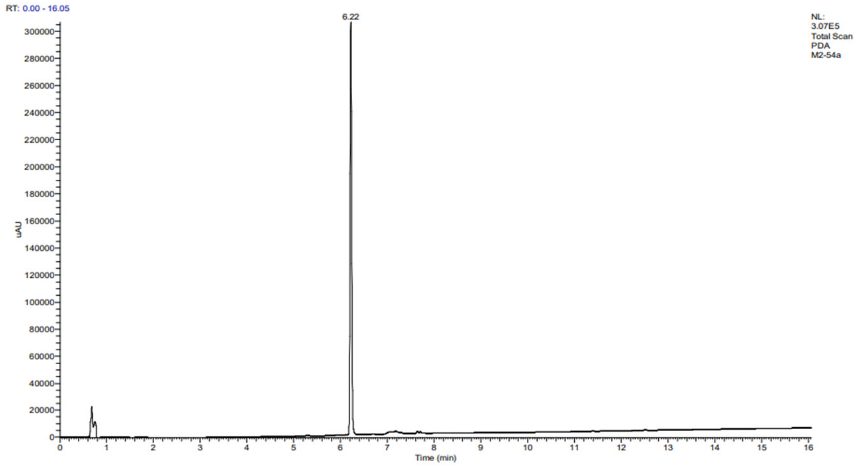

PEAK LIST
M2-54a.raw
RT: 0.00 - 16.05
Number of detected peaks: 2

Apex RT	Start RT	End RT	Area	%Area	Height	%Height
6.22	6.15	6.37	748810	90.08	305982	99.11
7.17	6.96	7.84	82492.3	9.92	2733.82	0.89

Figure S1: The LC-MS analysis of (−)-LRSL.

The structural information and LC-MS analysis of (–)-lariciresinol-4,4'-bis-*O*-*β*-D-glucopyranoside are as follows:

(–)-lariciresinol-4,4'-bis-*O*-*β*-D-glucopyranoside was obtained as white amorphous powder. ^{1}H NMR (DMSO-d_6, 600 MHz) δ: 7.01 (d, J = 8.4 Hz, 1H), 6.97 (d, J = 8.4 Hz, 1H), 6.88 (d, J = 1.8 Hz, 1H), 6.81 (d, J = 1.8 Hz, 1H), 6.77 (dd, J = 8.4, 1.8 Hz, 1H), 6.67 (dd, J = 8.4, 1.8 Hz, 1H), 4.85 (d, J = 7.2 Hz, 1H), 4.82 (d, J = 7.2 Hz, 1H), 4.71 (d, J = 4.8 Hz, 1H), 3.89 (t, J = 7.2 Hz, 1H), 3.74 (s, 6H), 3.69–3.64 (m, 3H), 3.56 (t, J = 7.2 Hz, 1H), 3.49-3.42 (m, 3H), 3.29-3.23 (m, 6H), 3.17-3.13 (m, 2H), 2.84 (dd, J = 13.8, 4.2 Hz, 1H), 2.60 (m, 1H), 2.48 (m, 1H), 2.20 (t, J = 7.2 Hz, 1H); HRMS: calcd for $C_{32}H_{44}O_{16}Na$ [M + Na]$^+$: 707.2525, found: 707.2522.

Thermo Qexactive Focus Report

compound **wl-6-14-t3b**
Method : **LCMS(compou**

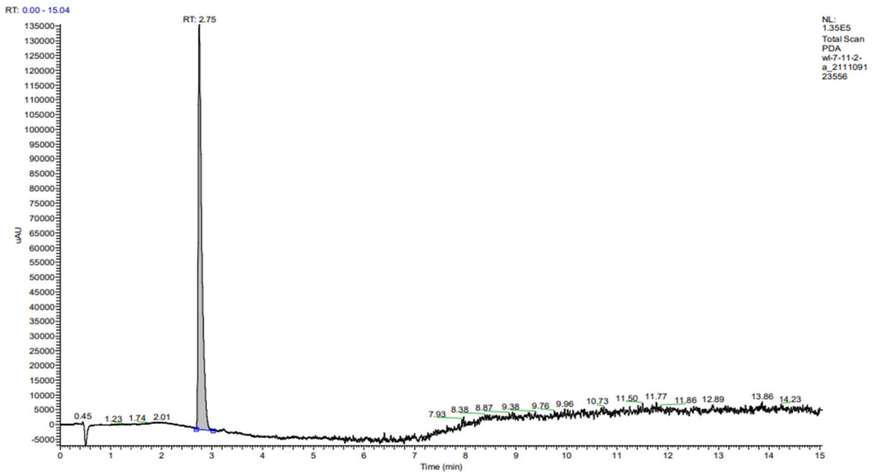

PEAK LIST
wl-7-11-2-a_211109123556.raw
RT: 0.00 - 15.04
Number of detected peaks: 1

Apex RT	Start RT	End RT	Area	%Area	Height	%Height
2.75	2.68	3.02	670994	100	137130	100

Figure S2: The LC-MS analysis of (–)-lariciresinol-4,4'-bis-*O*-*β*-D-glucopyranoside.

The structural information and LC-MS analysis of (–)-lariciresinol-4-*O*-*β*-D-glucopyranoside are as follows:

(–)-lariciresinol-4-*O*-*β*-D-glucopyranoside was obtained as white amorphous powder. ^{1}H NMR (DMSO-d_6, 500 MHz) δ: 8.67 (s, 1H,), 7.01 (d, *J* = 8.4 Hz, 1H), 6.88 (s, 1H), 6.77 (dd, *J* = 8.4, 1.8 Hz, 1H), 6.73 (s, 1H), 6.66 (d, *J* = 7.8 Hz, 1H), 6.56 (d, *J* = 7.8 Hz, 1H), 4.85 (d, *J* =7.0Hz, 1H) , 3.88 (dd, *J* = 7.5, 6.5 Hz, 1H), 3.69–3.64 (m, 2H), 3.56 (dd, *J* = 8.0, 7.0Hz, 1H), 3.50–3.40 (m, 2H), 3.27-3.20 (m, 3H), 3.16 (m, 1H), 2.80 (dd, *J* = 13.5, 4.5 Hz, 1 H) , 2.60 (m, 1H), 2.57 (m, 1H) , 2.16 (m, 1H); HRMS: calcd for $C_{26}H_{33}O_{11}$ [M – H]$^-$: 521.2031, found: 521.2028.

Thermo Qexactive Focus Report

compound **wl-6-14-t3a**
Method : **LCMS(compou**

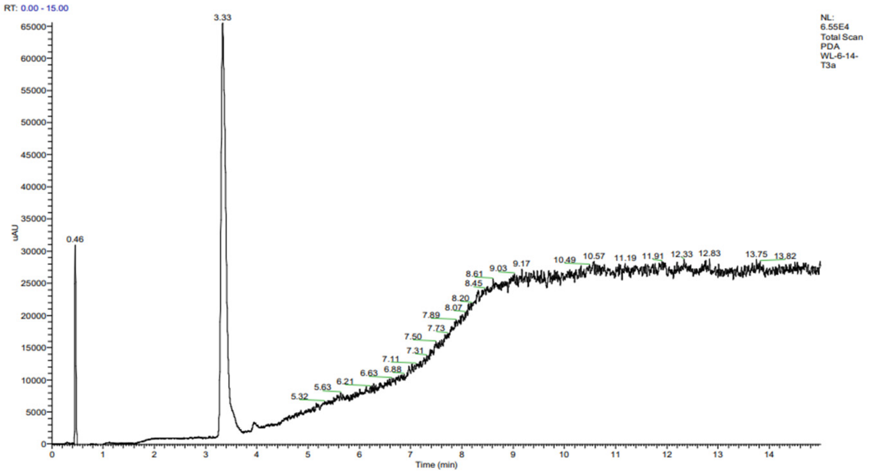

PEAK LIST
WL-6-14-T3a.raw
RT: 0.00 - 15.00
Number of detected peaks: 2

Apex RT	Start RT	End RT	Area	%Area	Height	%Height
3.33	3.2	3.69	445233	97.56	64322.3	97.67
3.95	3.89	4.13	11130.3	2.44	1537.49	2.33

Figure S3: The LC-MS analysis of (–)-lariciresinol-4-*O*-*β*-D-glucopyranoside.

The structural information and LC-MS analysis of lariciresinol-4'-*O*-*β*-D-glucopyranoside are as follows:

lariciresinol-4'-*O*-*β*-D-glucopyranoside was obtained as white amorphous powder. ^{1}H NMR (CD_3OD, 500 MHz) δ 6.93 (d, J = 2.0 Hz, 1H), 7.07 (d, J = 8.5 Hz, 1H), 6.82 (dd, J = 8.5, 2.0 Hz, 1H), 4.77 (m, 1H), 2.29 (m, 1H), 3.77 (1H), 3.59 (dd, J = 11.0, 7.0 Hz, 1H), 6.73 (d, J = 2.0 Hz, 1H), 6.65 (d, J = 8.0 Hz, 1H), 6.58 (dd, J = 8.0, 2.0 Hz, 1H), 2.85 (dd, J = 13.5, 5.0 Hz, 1H), 2.44 (dd, J = 13.5, 9.0 Hz, 1H), 2.66 (m, 1H), 3.94 (dd, J = 8.0, 6.5 Hz, 1H), 3.68 (dd, J = 8.0, 6.0 Hz, 1H), 4.82 (d, J = 7.0 Hz, 1H), 3.38-3.45 (m, 2H), 3.31-3.37 (m, 2H), 3.81 (1H), 3.63 (m, 1H), 3.80 (3H), 3.77 (1H); HRMS: calcd for $C_{26}H_{33}O_{11}$ $[M - H]^-$: 521.2032, found: 521.2028.

Thermo Qexactive Focus Report

compound **wl-6-14-t3b**
Method : **LCMS(compou**

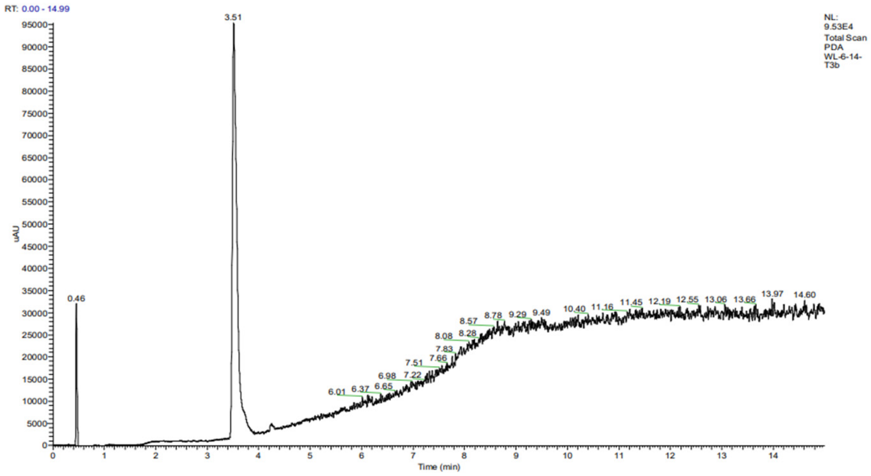

PEAK LIST
WL-6-14-T3b.raw
RT: 0.00 - 14.99
Number of detected peaks: 2

Apex RT	Start RT	End RT	Area	%Area	Height	%Height
3.51	3.42	3.92	599184	98.16	93677	97.69
4.25	4.21	4.36	11221.3	1.84	2214.02	2.31

Figure S4: The LC-MS analysis of lariciresinol-4'-*O*-*β*-D-glucopyranoside.
